# Safety and efficacy of radial access versus femoral access for rotational atherectomy: an updated systematic review and meta-analysis

**DOI:** 10.1186/s13019-025-03512-9

**Published:** 2025-06-21

**Authors:** Muhammad Ahmed, Muzna Murtaza, Muhammad Muzammil, Syeda Zuha Sami, Ariba Nazir, Muhammad Ahmed, Afsana Ansari Shaik, Muhammad Sohaib Asghar

**Affiliations:** 1 Department of Internal Medicine, Shaheed Mohtarma Benazir Bhutto Medical College Lyari, Karachi, Pakistan; 2 Division of Nephrology and Hypertension, Mayo Clinic, Rochester, MN USA; 3 Department of Internal Medicine, AdventHealth, Sebring, FL USA

**Keywords:** Rotational Atherectomy, Radial, Femoral, Meta-Analysis

## Abstract

**Background:**

Rotational atherectomy has been performed using both radial and femoral access over the years, but there is a lack of consensus on the safety and efficacy of these access sites.

**Methods:**

PubMed, Google Scholar, and Cochrane Library were searched until May 2024 for studies comparing the radial and femoral approaches in patients undergoing rotational atherectomy. The primary outcome was major vascular site bleeding. Secondary outcomes included short-term mortality, long-term mortality, myocardial infarction, major adverse cardiovascular events (MACE), acute stent thrombosis, procedural success, procedural time, and hospital stay. Generic inverse variance (GIV) was used to pool the risk ratio for dichotomous outcomes and mean difference (MD) for the continuous outcomes, with corresponding 95% confidence intervals (CIs).

**Results:**

Twelve studies including 15,700 patients with a mean age of 77.77 years in the radial group and 74.04 years in the femoral group, who had undergone rotational atherectomy, were included in the analysis. For the outcome of major vascular site bleeding, there was a significantly lower risk (RR: 0.23; 95% CI [0.12, 0.41]; *p* < 0.00001) in the radial group as compared to the femoral group. From the secondary outcomes, radial access was found to have significantly lower MACE (RR:0.80; 95% CI [0.68, 0.93]; *p* = 0.004), shorter procedural time (MD: -6.95; 95% CI [-11.52, -2.38], p = 0.003) and hospital stay (MD: -2.8; 95% CI [-5.56, -0.04], *p* = 0.05) as compared to femoral group. In contrast, all the other secondary outcomes were found to be insignificant.

**Conclusion:**

Rotational atherectomy using the radial approach has a significantly lower rate of major vascular site bleeding and MACE and is associated with significantly shorter procedural time and hospital stay.

**Supplementary Information:**

The online version contains supplementary material available at 10.1186/s13019-025-03512-9.

## Introduction

Rotational atherectomy is employed in circumstances where the plain old balloon angioplasty is unsuccessful and heavily calcified lesions persist via the removal of plaque or debulking rather than rupturing and compressing coronary atheroma and to aid in the deployment of stents in severely calcified lesions. [1, 2] One of the atherectomy devices involves High-Speed Rotational Atherectomy (HSRA) which is preferred due to its remarkable success rate and efficiency in treating profound calcified lesions. [3] Under local anesthesia a thin guidewire is cautiously inserted through the artery, thus providing access for a rotational atherectomy device. A specialized burr is inserted towards the calcified area and its precise rotation of about 150,000 to 200,000 rotations per minute (rpm) enables the cutting down and ablation of the deposited calcium, permitting the stent expansion and original blood flow restoration. [4] Rotational atherectomy is used in less than 5% of percutaneous coronary intervention (PCI) procedures and has a procedural success rate of 93.4%. [4, 5].

Trans-radial or transfemoral approaches can be used to execute rotational atherectomy. [6] Formerly, femoral access has been employed extensively due to the larger diameter of the femoral artery, and its easy palpability and accessibility however it was also associated with adverse effects like bleeding, occlusion, and prolonged recovery time. [7, 8] In the last decade, the radial artery has been more widely used. [9] and the use of the femoral approach has evolved to the radial approach as it is associated with increased patient comfort, lower health costs, and reduced complications like major adverse cardiovascular events (MACE) and bleeding are less seen with the trans-radial approach yet the uncertainty persist as the radial artery cannot accommodate large-size catheters and spasms occurs frequently. [8, 10].

To address these uncertainties, we conducted an updated systematic review and meta-analysis, incorporating a larger sample size and applying rigorous statistical methods, including a comprehensive meta-regression, to evaluate the impact and reliability of trans-radial and transfemoral access in rotational atherectomy. While randomized controlled trials (RCTs) remain the gold standard, they are often limited by ethical, logistical, and financial challenges. Observational studies offer valuable real-world data on procedural outcomes, and meta-analyses improve statistical power while minimizing variability. This study integrates existing evidence to compare radial and femoral access in rotational atherectomy, emphasizing key findings and identifying areas for future research, including the need for well-designed RCTs.

## Methods

This meta-analysis followed the Preferred Reporting Items for Systemic Review and Meta-analysis (PRISMA) guidelines. [11].

### Data sources and search strategy

A comprehensive literature search was conducted on PubMed, Google Scholar, and Cochrane Library databases from inception to May 2024. Online databases such as www.clinicaltrials.gov, medRxiv.org, and conference proceedings and presentations were also searched to identify grey literature, and bibliographies of relevant articles were also searched to make sure that no studies were missed. No limitations of age, language, time, or sample size were applied. The following keywords and their MeSH terms were used in the thorough literature search: “radial access”, “femoral access”, and “rotational atherectomy”. Detailed search strategies used in these databases are given in the supplementary material (**Table S1**).

### Study selection and characteristics

All articles retrieved from the databases were transferred to Endnote X9 (Clarivate Analytics, US), to remove the duplicated articles. Two individual reviewers (M.A and M.M) carefully screened the remaining articles, first by title and abstract, and then full-text articles were reviewed. In case of any conflict, a third reviewer (S.Z.S) was consulted. Studies were selected on the following inclusion criteria: a) randomized controlled trials and observational studies that compared radial access vs femoral access site in patients who underwent rotational atherectomy, b) patients above 18 years, and c) studies that reported at least one outcome of interest. Outcomes of interest were reported which included: major vascular site bleeding which was defined as major bleeding according to the Bleeding Academic Research Consortium (BARC) of type 3 to type 5 to be considered major, short-term mortality which was defined as in-hospital mortality or 30-day mortality, long-term mortality which was defined as death reported beyond 30 days, myocardial infarction (MI), MACE, acute stent thrombosis, procedural success, procedural time, hospital stay and radiation exposure. MACE was defined variably across the included studies. While some studies considered MACE as a composite of death, myocardial infarction, and target vessel revascularization, others included stroke, urgent coronary artery bypass grafting, or all-cause mortality. Desta et al. defined MACE as death, MI, or TVR, while Kotowycz et al. included urgent CABG. Kubler et al. considered all-cause mortality, follow-up MI, and stroke, whereas Watt et al. included cerebrovascular events. Yin et al. further expanded MACE to recurrent non-fatal MI and stroke. Baseline characteristics of the included studies and patients are summarized in Table 1 and Table S2. Angiographic data of patients in the included studies is summarized in Table S3.

**Table 1:** General characteristics of included studies

Study	Study design	Number of Participants, n		Male, (%)		Age (years), mean (SD)/Median (IQR)	
		RA	FA	RA	FA	RA	FA
Dall’ara 2023 [22]	Cohort	114* 37**	72	80.7* 62.2**	65.3	74 (9.2) * 74.7 (8.4) **	74.8 (9.1)
Desta 2022 [20]	Cohort	649	782	71.3	70.3	72.3 (9.2)	72 (9.3)
Ferstl 2022 [21]	Cohort	171	256	83.60	72.70	76 (69–81)	75 (67–81)
Giustino 2023 [27]	Cohort	326	1890	-	-	69.4 (10.1)	70.3 (10.4)
Januszek 2020 [23]	Cohort	855	855	67.1	66.2	71.57 (9.56)	91.98 (9.73)
Kassimis 2014 [24]	Cohort	60	75	70	74	74.9 (8)	74.6 (10)
Kotowycz 2015 [25]	Cohort	52	67	83	64	71 (10.2)	71.3 (10.5)
Kubler 2018 [14]	Cohort	123	54	73	57	71 (9)	72 (10)
Solomonica 2020 [28]	Cohort	190	173	68	67	68.4 (13.1)	72.4 (10.2)
Watt 2009 [26]	Cohort	75	76	73.3	59.2	68.2 (8)	68.2 (10)
Watt 2017 *** [12]	Cohort	3069	5553	75.1	70.3	72.5 (0.17)	73 (0.12)
Yin 2015 [13]	Cohort	59	67	71	51	69.3 (1.3)	72.9 (1.6)

FA, femoral access; IQR, interquartile range; RA, radial access; SD, standard deviation

^*^radial standard access, **radial sheathless guiding catheter access, ***Patient totals are delineated individually for each characteristic due to incomplete data retrieval

### Data extraction and quality assessment

Two reviewers (M.M and M.M) conducted the data extraction of selected studies. The following data was extracted from each study: a) study name and year, b) study design, c) mean age of patients, d) number of patients in each group (radial access versus femoral access), and e) outcomes of interest. To address potential biases in observational data, we conducted a quality assessment using the Newcastle–Ottawa Scale (NOS) and AMSTAR 2 tool. [15] The AMSTAR 2 tool is utilized for assessing methodological quality, ensuring that the conclusions and findings are rooted in the utmost quality evidence. Figure S2 [16].

### Statistical analysis

For statistical analysis, Review Manager (RevMan Version 5.4.1) provided by Cochrane Collaboration Network was used. Dichotomous data was used to derive the risk ratio (RR) and corresponding 95% confidence interval (95% CI). Generic inverse variance (GIV) was used to pool the risk ratio and corresponding 95% CI. A random-effects model was used to pool the primary and secondary outcomes, and their results are presented as forest plots. Higgins I^2^ was used to measure heterogeneity. [17, 18] The value of I^2^ = 25–50% was considered mild heterogeneity, 50–75% was considered moderate, and greater than 75% was considered severe heterogeneity. A p-value ≤ 0.05 was considered statistically significant. Funnel plots were constructed for the outcomes which included more than 10 studies to check for any publication bias. Comprehensive Meta-Analysis (CMA) (Version 3.3.070) was used to perform meta-regression to explore sources of heterogeneity and funnel plots were used to assess publication bias. These methodological approaches enhance the reliability of our findings and help minimize the impact of confounding factors. [19].

## Results

### Study selection and characteristics.

A comprehensive literature search was conducted and yielded 847 articles. Upon removing ineligible, duplicates, case report articles. A total of 12 studies [16–27] which were observational and were included in this meta-analysis. The PRISMA flowchart presents the summary of the literature search (Figure S1). The number of patients from these 12 studies totaled 15,700 (5,780 in the radial group versus 9,920 in the femoral group). The follow-up time was 1211.8 days. The mean age of patients in the radial group was 77.77 years and 74.04 years in the femoral group. The baseline characteristics of the included studies are presented in Table 1, and the baseline characteristics of patients are summarized in Table S2. Angiographic data of patients of the included studies is summarized in Table S3. Detailed data on sheath sizes used in radial and femoral access Table S4 and the anticoagulant and antiplatelet strategies employed across included studies Table S5.

### Primary outcome

#### Major vascular site bleeding

**Fig. 1:**
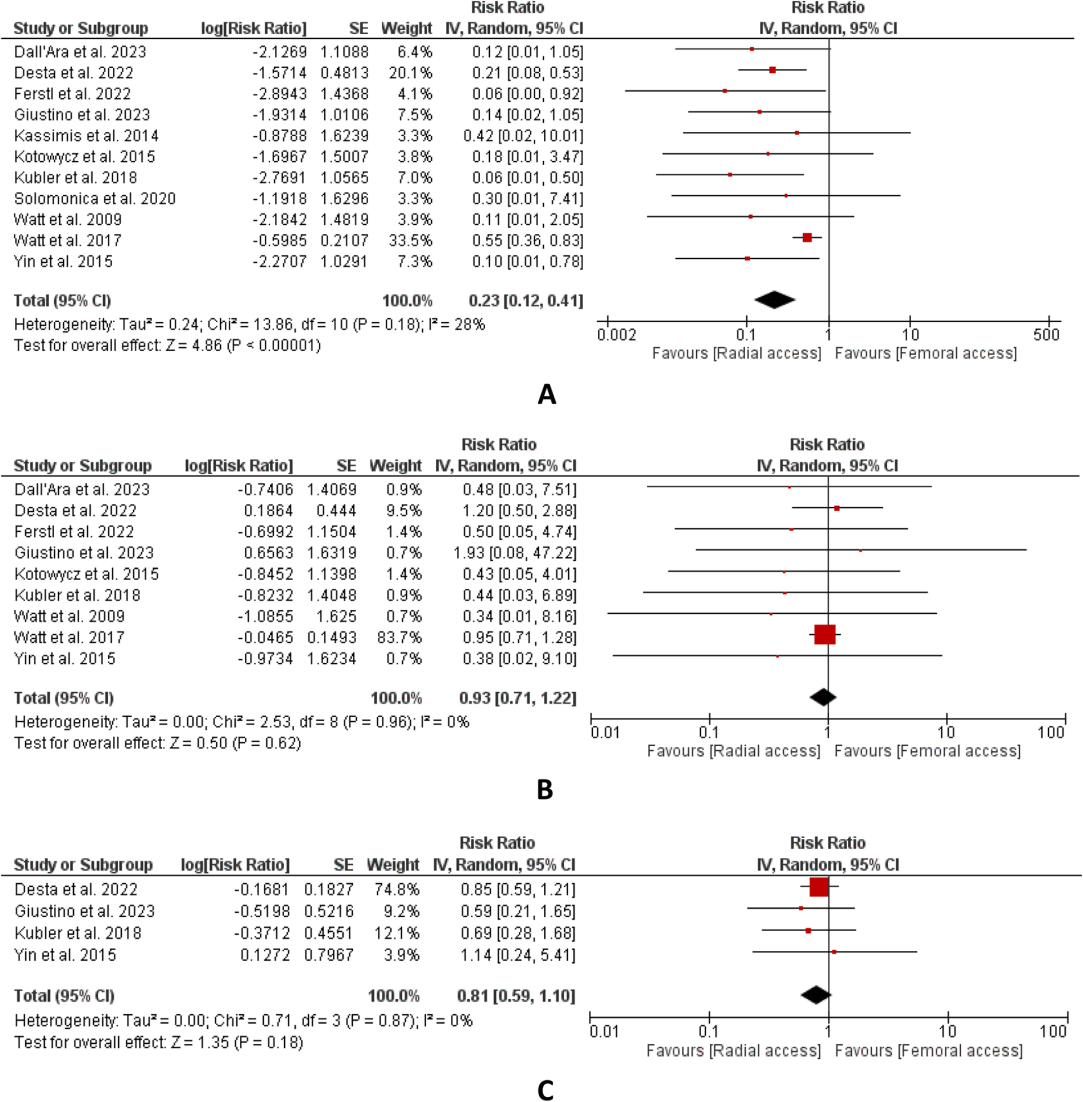
**A** major vascular site bleeding, **B** short-term mortality, **C** long-term mortality

Our meta-analysis of 11 studies showed that there were significantly lower number of events of major vascular bleeding in the radial group as compared to the femoral group. The overall results were statistically significant. (RR:0.23, CI: [0.12,0.41]; P < 0.00001, I^2^ = 28%) (Fig. 1A).

### Secondary outcomes

**Fig. 2:**
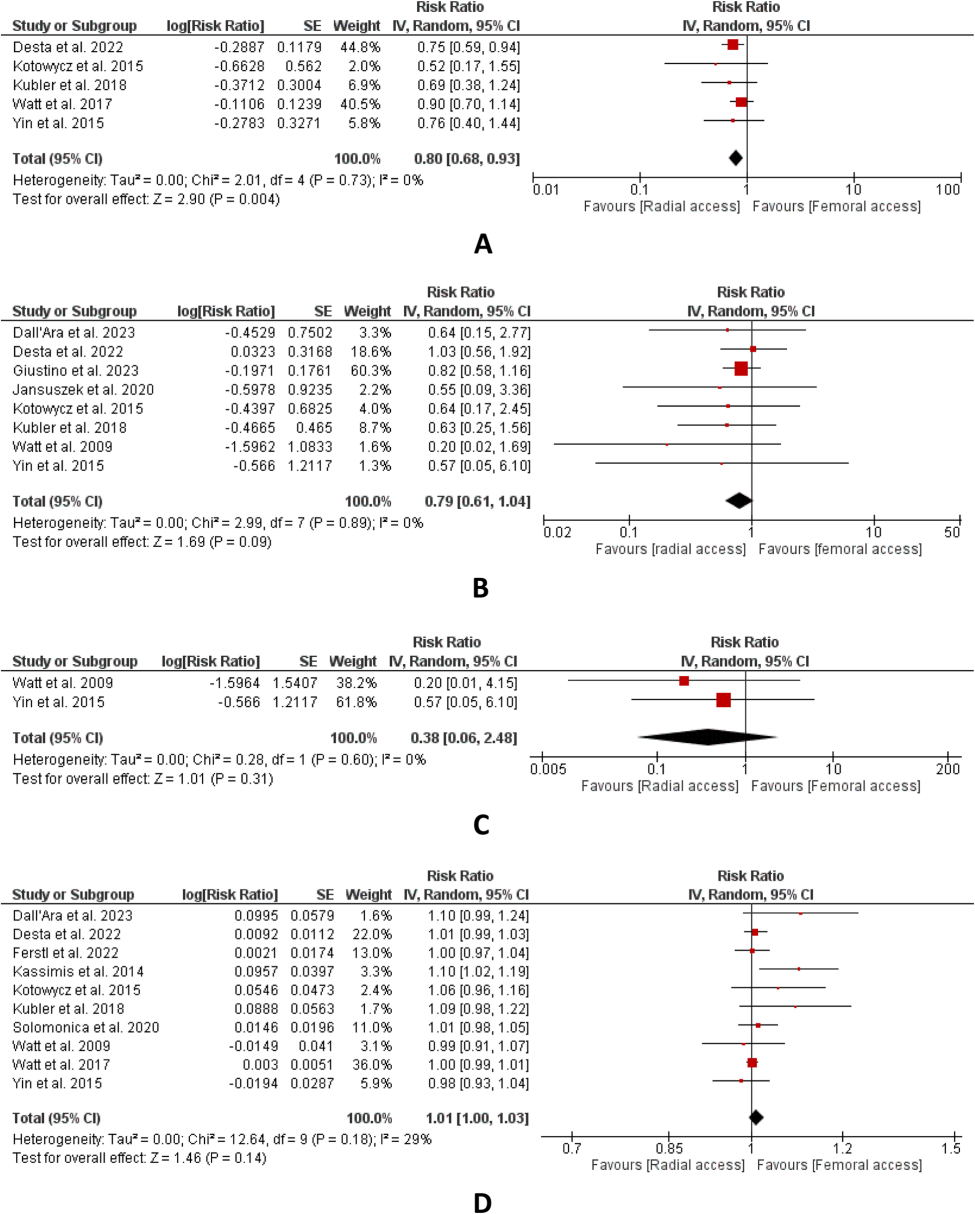
**A** MACE, **B** MI, **C** acute stent thrombosis, **D** procedural success

**Fig. 3:**
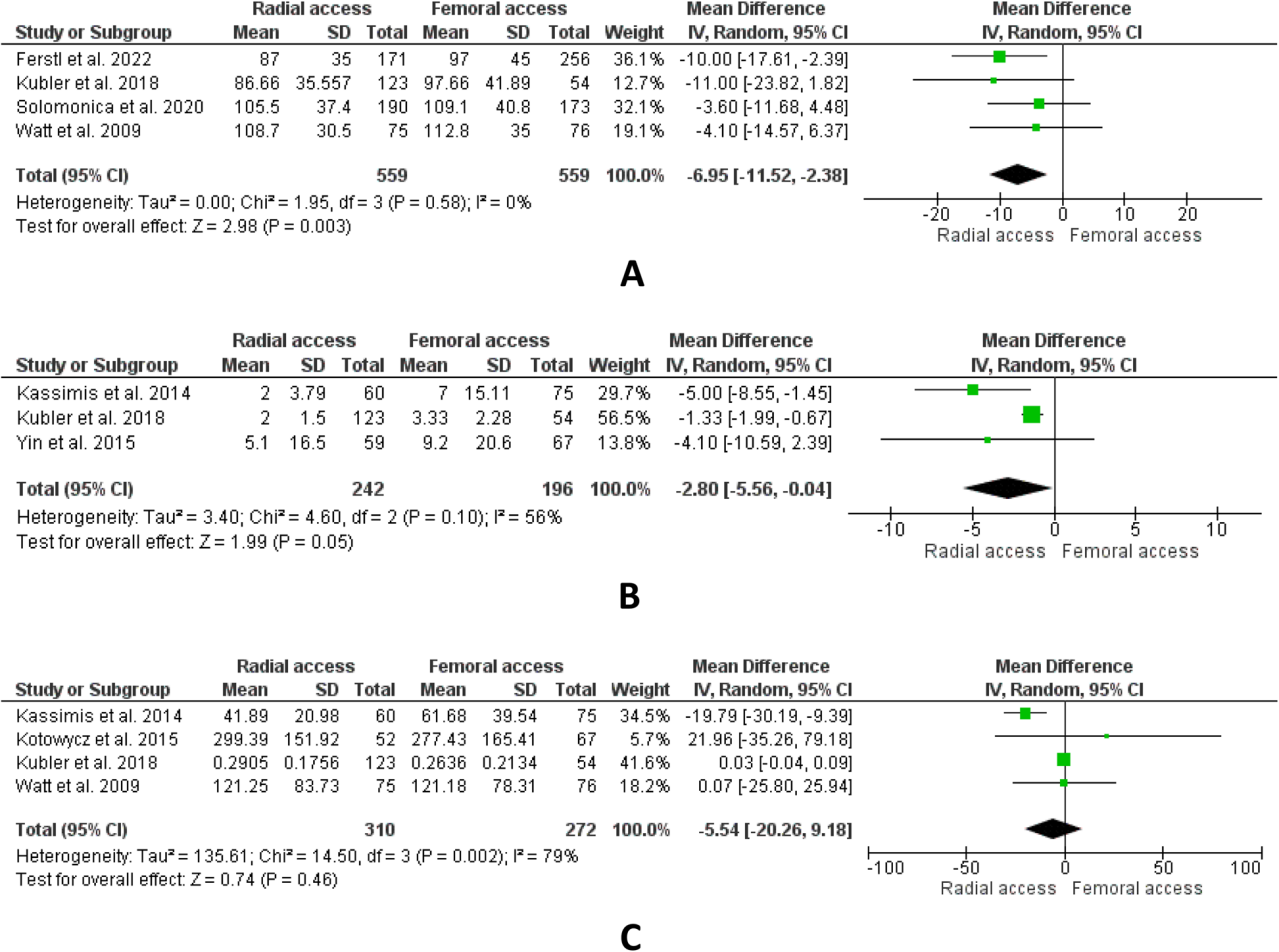
**A** procedural time, **B** hospital stay, **C** radiation exposure

A total of nine studies reporting short-term mortality declared overall results statistically insignificant. (RR:0.93, 95% CI: [0.71,1.22]; *P* = 0.62; I^2^ = 0%) (Fig. 1B). In the outcome of long-term mortality, four studies were included. The result of combined findings revealed statistical insignificance. (RR:0.81, CI: [0.59,1.10]; *P* = 0.18, I^2^ = 0%) (Fig. 1C). Five studies that reported MACE were included and the results indicated that this outcome has a lower number of events in the radial group. The overall results were statistically significant. (RR:0.80, CI: [0.68,0.93]; P = 0.004, I^2^ = 0%) (Fig. 2A). Eight studies in total reported MI incidence. The results of combined findings revealed statistical insignificance. (RR:0.79, CI: [0.61,1.04]; *P* = 0.09, I^2^ = 0%) (Fig. 2B). A total of two studies documenting the outcome of acute stent thrombosis were combined for analysis. The result revealed statistical insignificance between both groups. (RR:0.38, CI: [0.06,2.48]; P = 0.31, I^2^ = 0%) (Fig. 2C). A total of ten studies reported procedural success indicating no statistical difference between both groups for this outcome and the result is found to be insignificant (RR:1.01, CI: [1.00,1.03]; *P* = 0.14, I^2^ = 29%) (Fig. 2D). For the outcome of procedural time, four studies were included. Upon aggregation, the combined analysis revealed shorter procedural time in the radial group and overall results were statistically significant. (MD: −6.95, CI: [−11.52, −2.38]; P = 0.003, I^2^ = 0%) (Fig. 3A). Three studies reported hospital stays in which the results demonstrated statistical significance. The findings revealed shorter hospital stays in the radial group as compared to the femoral group. (MD: −2.8, CI: [−5.56, −0.04]; P = 0.05, I^2^ = 56%) (Fig. 3B). In the outcome of radiation exposure, four studies were included, and the overall results were found to be statistically insignificant. (MD: −5.54, CI: [−20.26,9.18]; P = 0.46, I^2^ = 79%) (Fig. 3C).

#### Assessment of heterogeneity

Sensitivity analysis which removed one study at a time revealed that after eliminating Kassismis et al. 2014, which was found to be an outlier study, the substantial heterogeneity in the overall estimate decreased from 79 to 0% and the results remained insignificant. This helped to explain the high heterogeneity in the plot of radiation exposure. (RR:0.03, CI: [−0.04,0.09]; P = 0.42, I^2^ = 0%) (Figure S3).

### Meta-regression

We performed a meta-regression analysis to assess various factors that could influence the effect size on our primary outcome: major vascular site bleeding. The covariates examined included mean age, percentage of male participants, hypertension, diabetes mellitus, smoking, BMI, hyperlipidemia, prior myocardial infarction (MI), previous coronary artery bypass grafting (CABG), ejection fraction, previous peripheral artery disease (PAD), and previous kidney disease. The findings revealed significant associations between major vascular site bleeding and the percentage of male participants, prior MI, and previous PAD. In the meta-regression analysis, male sex (%) was a significant predictor (coefficient: 0.1984, p = 0.0050), while prior MI (coefficient: −0.0832, p = 0.0226) and previous PAD (coefficient: −0.0883, p = 0.0195) were also identified as significant factors. Table 2 presents the coefficients and p-values for each covariate assessed in the analysis. Scatter plots for all factors assessed in the meta-regression are included in Figure S5-S8.

**Table 2:** Meta-regression analysis results

Variables	Coefficient	*P*-value
Mean age	0.1229	0.5495
Male sex %	0.1984	0.0050
Hypertension	0.0514	0.5605
Diabetes mellitus	−0.0267	0.6151
Smoking	−0.0114	0.7148
Body-mass-index	0.3443	0.4573
Hyperlipidemia	0.0177	0.5896
Previous myocardial infarction	−0.0832	0.0226
Previous coronary artery bypass grafting	−0.1512	0.092
Ejection fraction	−0.0138	0.9404
Previous peripheral artery disease	−0.0883	0.0195
Previous kidney disease	0.0686	0.0949

### Quality assessment and publication bias

Quality assessment of 12 cohort studies was done using the Newcastle–Ottawa Scale (NOS). Cohorts have a score of ≥ 6 (Table S6). For the outcome, which consisted of at least 10 studies was assessed for publication bias using funnel plots (Figure S4). Funnel plots that showed asymmetry were further assessed for confirmation using Begg’s and Egger's tests. The p-value of the Beggs and Egger tests are provided in Table S7.

## Discussion

The purpose of our systematic review and meta-analysis of 12 published articles [16–27] was to compare the effectiveness and safety of radial access versus femoral access in rotational atherectomy. Given the limited availability of RCTs in this domain, observational data serve as a critical source of real-world evidence, allowing for a broader understanding of procedural outcomes, complications, and clinical applicability. While observational studies inherently carry risks of selection bias and confounding, rigorous statistical techniques such as meta-regression strengthen the validity of our findings. Our findings demonstrate that the radial technique is a safe and effective way to facilitate rotational atherectomy for severely calcified lesions. In comparison to femoral access, it is associated with significantly lower rates of major vascular site bleeding, fewer MACE, shorter procedural time and hospital stay, similar procedural success rates, and no appreciable differences in all-cause mortality, myocardial infarction occurrences, and acute stent thrombosis. Our meta-regression found that male sex increased the risk of major vascular site bleeding, while prior MI and PAD were linked to lower risk. This highlights the importance of optimizing vascular access strategies to minimize complications in high-risk patients.

Our findings align with those of an earlier meta-analysis that included five studies [12, 13, 24–26] with 9153 patients, which reported significantly lower major vascular site bleeding in the radial access group. This analysis was conducted by Khan in 2018. [29] By utilizing a larger sample size, our study builds on these findings, revealing that while procedural success was similar between groups, the radial access group also experienced significantly fewer MACE, shorter procedural times, and reduced hospital stays. This enhances the reliability of our results and supports the notion that radial access is a safer and more effective alternative to femoral access.

In the field of interventional cardiology, where patient outcomes are prioritized, vascular site bleeding is a crucial problem during rotational atherectomy. [29] The management of access site bleeding is greatly influenced by the anatomical position of both arteries. The femoral arteries are particularly difficult to achieve adequate hemostasis due to their bigger size, central position, being surrounded by soft structures, lack of firm bony support, and deep seating. [30–32] Conversely, the superficial placement and relatively smaller diameter of radial arteries facilitate easier compression of the artery against the radius bone, hence lowering the risk of bleeding. [33, 34] Furthermore, the tremendous blood volumes that traverse through and the comparatively minimal collateral blood supply make femoral artery bleeding more detrimental and may even result in hypovolemic shock. [34–37] While there are now effective hemostatic devices available such as Vascular Closure Devices (VCD), studies indicate that the transfemoral route still has higher access site complications, such as hematomas, delayed ambulation, and delayed discharge even with device use. [38] These benefits of lesser bleeding at the access site highlight the reasons radial access is becoming more and more preferred than femoral access, optimizing patient safety during rotational atherectomy. While radial access generally does not require vascular closure devices (VCDs), the choice of closure strategy in femoral access may influence bleeding outcomes. Some studies reported the use of closure devices such as Angio-Seal and Perclose, which could potentially reduce vascular complications.

Our analysis has uncovered new insights regarding procedural time, hospital stay, and MACE, which are now statistically significant compared to previous results. These findings further reinforce the idea that radial access is a superior route to femoral access. High risk of severe bleeding, which may occasionally be retroperitoneal hemorrhage and delayed ambulation in femoral access, which can lengthen bed rest periods and cause thrombotic events like deep-vein thrombosis (DVT), both of which can raise serious vascular problems and ultimately cause MACE. [39–41] Extended hospital stays can exacerbate anxiety and stress levels in patients, which may delay healing. [42] Additionally, research indicates that femoral access catheterization carries a higher risk of infection and colonization, which may contribute to a higher incidence of adverse cardiovascular events in this patient population compared to those with radial access. [43] Although radial access is a more complex procedure requiring an experienced operator, it is still feasible because the radial artery is easily accessible, easy to compress in the event of bleeding, and associated with fewer procedural complications, all of which support the outcome that radial access has a shorter turnaround time of the procedure. [44, 45] In our study, radial access revealed a numerical preference for the occurrences of lower events of myocardial infarction and acute stent thrombosis between the two groups, even though the results are not statistically significant. Acute stent thrombosis can occur from trauma to the artery during catheter placement, which exposes the endothelium and activates the clotting mechanism. [46] Moreover, prolonged immobilization and bed rest following the femoral access procedure also contribute to thrombosis occurrences. [40, 41] The blood supply to the heart muscles can eventually be compromised by excessive bleeding, more severe vascular site issues, and more thrombosis events in the femoral access, leading to major complications like myocardial infarction.

Our data also indicated numerically that patients with femoral access had higher all-cause mortality, although the results are not statistically significant. Our meta-analysis demonstrated no significant difference in all-cause mortality between radial and femoral access groups. Therefore, any claims suggesting a mortality benefit of one approach over the other are not supported by our findings. While radial access is associated with lower vascular complications, its impact on survival remains inconclusive. The aforementioned factors, such as increased major access site bleeding, retroperitoneal bleeding that may result in hypovolemic shock, severe cardiovascular adverse effects, and an increased risk of infection in the femoral artery, all worsen the patient's condition and ultimately cause death, suggesting that patients with femoral access have a higher risk of death than those with radial access. [47] Our data significantly suggested that patients with femoral access stay longer in hospitals, this is because, femoral access has a higher risk of complications and severe adverse effects, which must be closely monitored during a patient's hospital stay. [48] Parallel to the previous meta-analysis, we discovered that radiation exposure is lower in the radial access group than in the femoral access group; however, the results are not statistically significant, which can be attributed to the use of sophisticated techniques and increased operator experience and skills. [49] Our analysis further demonstrates that, regardless of the access point chosen for rotational atherectomy, procedural success was the same in both groups. Since the radial artery has a lesser diameter, a smaller burr and catheter is needed for the procedure. Research has demonstrated that, in comparison to larger burr sizes, small burr sizes (maximum of 1.5 mm burr and burr-to-artery ratio of 0.5) also yield comparable debulking and plaque modifying capability and success rates. Furthermore, for more complicated interventional procedures, a successful radial approach can be achieved with 6 Fr guide catheters. While both techniques yield similar procedural success, it is preferable to use radial access, as it is the one with fewer complications. [50] Our analysis highlights significant variability in sheath sizes between radial and femoral access, with radial procedures predominantly using 6 F sheaths, while femoral access frequently required 7 F or larger sheaths due to procedural demands.

### Future directions

Our findings demonstrated that, when it comes to safety and effectiveness, radial access is a considerably better approach than femoral access for rotational atherectomy procedures. However, Future studies could potentially explore several directions to enhance and broaden the relevance of these results. Firstly, more longitudinal research needs to be conducted to monitor long-term outcomes like mortality, the rate of restenosis, long-term adverse effects, and post-procedural quality of life. Secondly, advanced techniques should be used to assess any post-procedural vascular complications and effects. Furthermore, to ensure the strong validity of our observed findings, large-scale RCTs should be carried out. Although earlier research has indicated that radial access is more cost-effective, the fact that this approach has less bleeding and other complications also lends validity to the concept that it is a less expensive process. [51] To clarify the long-term advantages of radial access, further research should be done to demonstrate the cost-effectiveness of both access locations, particularly in the rotational atherectomy technique. In addition, there should be more practice with radial access to have many skilled and knowledgeable operators for the process. Radial access should be used whenever feasible by operators to lower the risk of complications and improve patient comfort, safety, and quality of life.

### Limitations

There were a few constraints to be aware of, despite adhering to the quality standards and following appropriate design guidelines when conducting our study. All the studies included are retrospective observational cohorts as RCTs were not available comparing radial access and femoral access for rotational atherectomy and more prospective and randomized studies would be preferable for future research as the non-randomized data is prone to confounding and selection biases. Also, the selection bias concerning different population demographics, ethnic diversity, operator experience, comorbid severity, and baseline differences could not be excluded. A limitation of our study is the inconsistent reporting of sheath sizes, with radial access primarily using 6 F and femoral access often requiring ≥ 7F. Larger sheaths in femoral access may increase vascular complications, while smaller sheaths in radial access reduce bleeding risk. Standardized sheath size reporting and evaluating the safety of limiting femoral sheaths to 6 F should be considered in future studies. Another limitation of our study is the inconsistency in anticoagulation and antiplatelet strategies across studies. While most used unfractionated heparin, some included bivalirudin or glycoprotein IIb/IIIa inhibitors, and ACT targets were not uniformly reported. Only a few studies met the criteria of using heparin alone with an activated clotting time (ACT) of 250–300 s, making direct comparisons challenging. Standardized anticoagulation protocols are needed for better assessment of procedural outcomes. One limitation of our study is the lack of consistent reporting on femoral vascular closure techniques across included studies. While some mentioned the use of closure devices, others did not specify their hemostasis methods, making it challenging to evaluate their effect on procedural outcomes. Future research should include detailed closure technique data to better compare VCD use with manual compression. Furthermore, we were unable to present long-term effects as in some trials the follow-up periods were short. Despite these limitations, we tried to uncover as many conclusive findings as we could to expand our understanding and present the most promising conclusions.

## Conclusion

Our study has provided evidence that radial access significantly takes less procedural time with shorter hospital stay duration, moreover, events of major vascular site bleeding and MACE are also significantly much lower in radial access procedures than in femoral access. Besides fewer procedural complications and adverse effects, it is found that procedural success is the same for both access site routes. Hence, radial access emerges as the superior, more efficient, and patient-friendly approach for rotational atherectomy than femoral access as it takes less procedural time and possesses fewer complications without compromising procedural success.

## Supplementary Information

Supplementary Material 1

## Data Availability

No datasets were generated or analysed during the current study.
