# Supplementary material for: Safety and efficacy of radial access versus femoral access for rotational atherectomy: an updated systematic review and meta-analysis
Source: J Cardiothorac Surg. 2025 Jun 21;20:266. doi: 10.1186/s13019-025-03512-9 (PMC12182652; doi:10.1186/s13019-025-03512-9)
Supplement: Supplementary file 1 — Supplementary Material 1 [file 13019_2025_3512_MOESM1_ESM.docx]

| PubMed (n=747) | (Transradial OR radial artery access OR TRA OR Transfemoral OR femoral artery access OR TFA) AND ("Rotational atherectomy" OR "RA" OR "Rotablation") |
| --- | --- |
| Google Scholar (n=2750) | (Radial approach OR Femoral approach) AND ("Rotational atherectomy") |
| Cochrane Library (n=93) | (Transradial OR radial artery access OR TRA OR Transfemoral OR femoral artery access OR TFA) AND ("Rotational atherectomy" OR "RA" OR "Rotablation") |

**Table S1.** Detailed search strategy used in each database.

| **Study** | **Hypertension, n (%)** | | **Diabetes mellitus, n (%)** | | **Current smokers, n (%)** | | **BMI (kg/m^2^), mean (SD)** | | **Hyperlipidemia, n (%)** | | **Prior myocardial infarction, n (%)** | |
| --- | --- | --- | --- | --- | --- | --- | --- | --- | --- | --- | --- | --- |
|  | RA | FA | RA | FA | RA | FA | RA | FA | RA | FA | RA | FA |
| **Dall’ara 2023** | 99 (86.8) *  32(86.5) ** | 62 (86.1) | 52 (45.6) *  13 (35.1) ** | 32 (44.4) | 37 (32.5) *  15 (40.5) ** | 28 (38.9) | - | - | 88 (77.8) *  26 (70.3) ** | 52 (72.2) | - | - |
| **Desta 2022** | 540 (83.2) | 633 (82) | 216 (33.5) | 287 (36.9) | 67 (10.7) | 68 (9.4) | 27.8 (8.2) | 27.5 (7.6) | 526 (81.1) | 648 (83.7) | 273 (42.9) | 387 (52.2) |
| **Ferstl 2022** | 156 (91.2) | 255 (95.7) | 50 (29.2) | 100 (39.2) | - | - | 27.4 (5) | 27.8 (4) | 158 (92.3) | 273 (92.5) | - | - |
| **Giustino 2023** | - | - | - | - | - | - | - | - | - | - | - | - |
| **Januszek 2020** | 628 (74.3) | 620 (14.9) | 272 (32.2) | 269 (31.8) | 144 (17) | 133 (15.7) | - | - | - | - | 436 (51.6) | 439 (52) |
| **Kassimis 2014** | 48 (80) | 66 (88) | 17 (28) | 24 (32) | 6 (10) | 8 (11) | 27 (5) | 30 (10) | 42 (70) | 49 (66) | - | - |
| **Kotowycz 2015** | 41 (79) | 58 (87) | 15 (29) | 33 (49) | - | - | - | - | 44 (85) | 58 (87) | 12 (23) | 25 (37) |
| **Kubler 2018** | 103 (84) | 44 (81) | 52 (42) | 26 (48) | 12 (10) | 2 (4) | - | - | 59 (48) | 22 (41) | 77 (63) | 34 (63) |
| **Solomonica 2020** | 108 (57) | 134 (78) | 61 (32) | 71 (41) | - | - | - | - | 88 (46) | 124 (72) | 50 (26) | 59 (34) |
| **Watt 2009** | 50 (66.7) | 49 (64.5) | 17 (22.7) | 21 (27.6) | 43 (57.3) | 47 (61.8) | 27.9 (4.3) | 26.8 (4.1) | 51 (68) | 55 (72.4) | 36 (48) | 36 (47.4) |
| **Watt 2017 ***** | 2129 (72.5) | 3917 (74.9) | 911 (30.4) | 1604 (30.2) | - | - | - | - | 2010 (68.6) | 3698 (70.8) | 1072 (37.6) | 2125 (42.3) |
| **Yin 2015** | 43 (73) | 56 (84) | 30 (50) | 38 (57) | 6 (10) | 8 (12) | 25.6 (6.5) | 25.1 (4.8) | 35 (59) | 41 (61) | 6 (10) | 11 (16) |

**CONTINUED ON THE NEXT PAGE**

| **Study** | **Prior PCI, n (%)** | | **Prior CABG, n (%)** | | **Ejection fraction (%), mean (SD), or Median (IQR)** | | **Prior CVA, n (%)** | | **Atrial fibrillation, n (%)** | | **PAD, n (%)** | | **Previous kidney disease, n (%)** | |
| --- | --- | --- | --- | --- | --- | --- | --- | --- | --- | --- | --- | --- | --- | --- |
|  | **RA** | **FA** | **RA** | **FA** | **RA** | **FA** | **RA** | **FA** | **RA** | **FA** | **RA** | **FA** | **RA** | **FA** |
| **Dall’Ara 2023** | - | - | - | - | 50 (45-55) *  50 (40-60) ** | 50 (42-55) | - | - | - | - | 35 (30.7) *  13 (35.1) ** | 30 (41.7) | 6 (5.3) * | 7 (9.7) |
| **Desta 2022** | 319 (49.2) | 449 (57.5) | 118 (18.2) | 198 (25.3) | - | - | - | - | - | - | - | - | - | - |
| **Ferstl 2022** | 73 (42.7) | 135 (52.9) | 17 (9.9) | 80 (31.3) | 51 (11) | 48 (14) | 14 (8.2) | 21 (8.3) | 39 (22.80) | 77 (29.90) | 26 (15.20) | 40 (15.70) | - | - |
| **Giustino 2023** | - | - | - | - | 57.1 (8.3) | 55.8 (9.2) | - | - | - | - | - | - | - | - |
| **Januszek 2020** | 486 (57.5) | 485 (57.4) | 124 (14.7) | 129 (15.3) | - | - | 40 (4.7) | 33 (3.9) | - | - | - | - | 115 (13.6) | 126 (14.9) |
| **Kassimis 2014** | - | - | - | - | 47 (10) | 48.5 (9.5) | - | - | - | - | - | - | 9 (8) | 13 (18) |
| **Kotowycz 2015** | 21 (40) | 27 (40) | 9 (17) | 11 (16) | - | - | 3 (6) | 3 (4) | - | - | - | - | - | - |
| **Kubler 2018** | 93 (76) | 38 (70) | 14 (11) | 11 (20) | 55 (40-60) | 55 (40-60) | 14 (11) | 7 (13) | 21 (17) | 14 (26) | 39 (32) | 15 (28) | - | - |
| **Solomonica 2020** | 32 (17) | 49 (28) | 25 (13) | 54 (31) | 52.7 (10.2) | 49.3 (12.1) | 16 (8.3) | 27 (16) | - | - | 21 (11) | 17 (9.9) | 18 (22) | 34 (21) |
| **Watt 2009** | 12 (16) | 15 (19.7) | 12 (16) | 16 (21.1) | - | - | 8 (10.7) | 11 (14.5) | - | - | - | - | - | - |
| **Watt 2017***** | - | - | 349 (11.6) | 964 (17.8) | - | - | 245 (8.4) | 373 (7.1) | - | - | 369 (12.6) | 603 (11.6) | 139 (4.8) | 418 (8.0) |
| **Yin 2015** | - | - | - | - | - | - | 4 (7) | 9 (13) | - | - | - | - | 14 (24) | 19 (29) |

BMI, body mass index; CABG, coronary artery bypass grafting; CVA, cerebrovascular accident; FA, femoral access; IQR, interquartile range; MI, myocardial infarction; PAD, peripheral artery disease; PCI, percutaneous coronary intervention; RA, radial access; SD, standard deviation

*radial standard access, **radial sheathless guiding catheter access, ***Patient totals are delineated individually for each characteristic due to incomplete data retrieval

**Table S2. Baseline characteristics of patients**

**Previous studies**

**Identification of new studies via databases and registers**

Studies included in previous version of review (n = 5)

Reports of studies included in previous version of review (n = 5)

Records identified from databases* (n=3590)

PubMed (n = 747)

Google Scholar (n = 2750)

Cochrane Library (n=93)

Records removed *before screening* (n=2743)

Duplicate records removed.

(n = 2743)

**Identification**

Total studies included in review.

(n = 12)

Reports of total included studies

(n = 12)

Reports assessed for eligibility.

(n = 20)

Reports sought for retrieval.

(n =49)

Records screened.

(n =847)

Records excluded.

(n =798)

Reports not retrieved.

(n =29)

**Screening**

Reports excluded (n=13)

No outcome of interest (n=8)

Non-comparative groups (n =5)

New studies included in review.

(n = 7)

Reports of new included studies

(n =7)

**Included**

**Figure S1.** PRISMA flowchart

**Figure S2.** AMSTAR 2 checklist


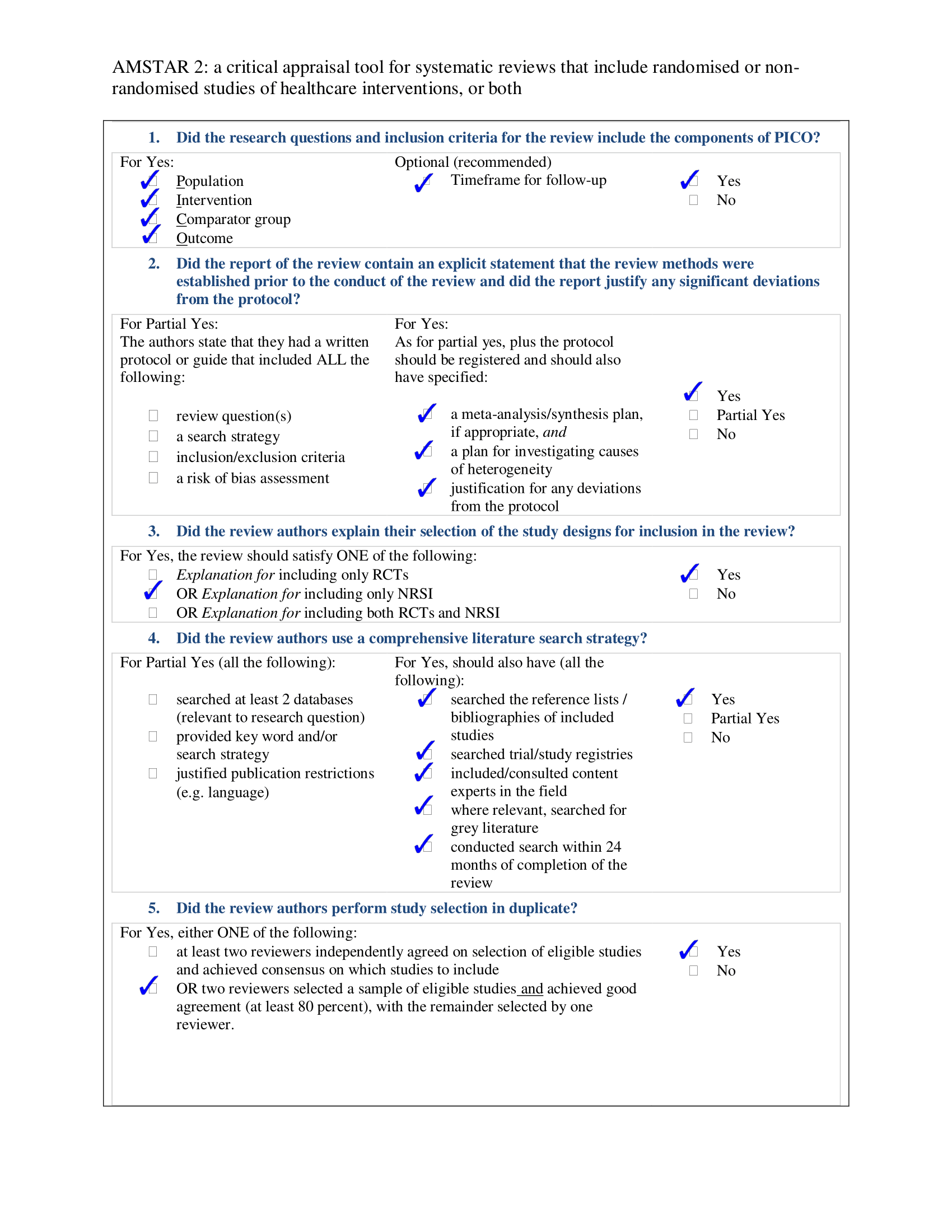


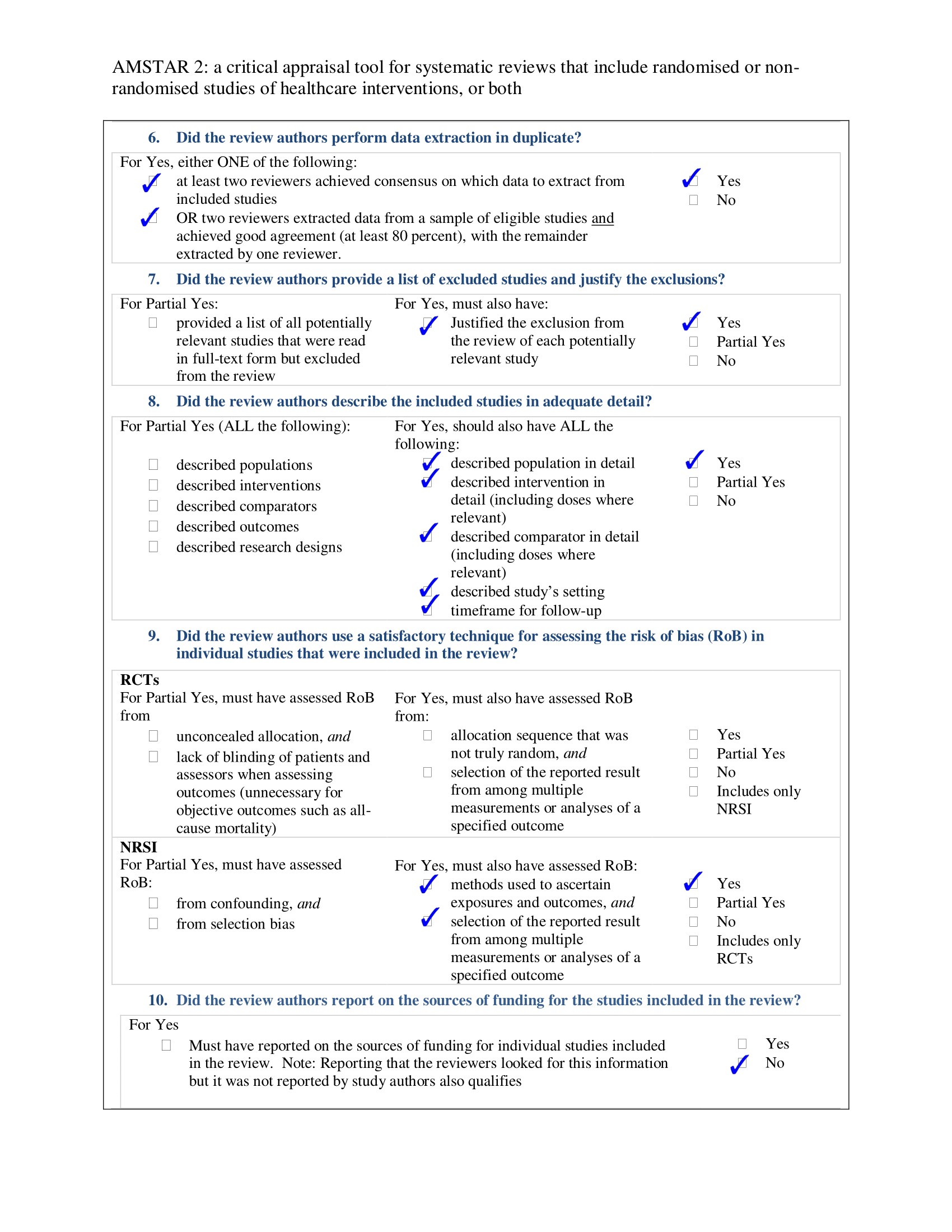


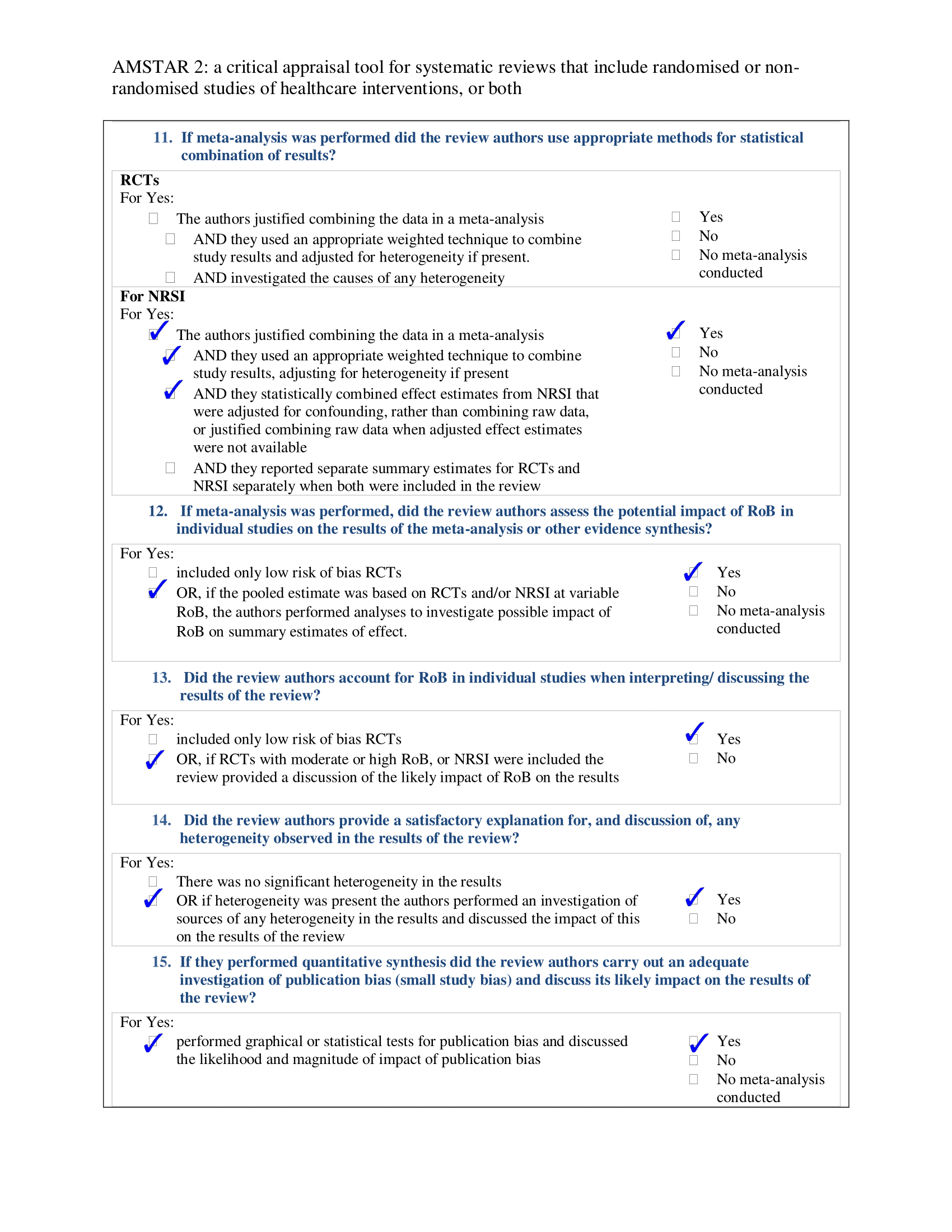


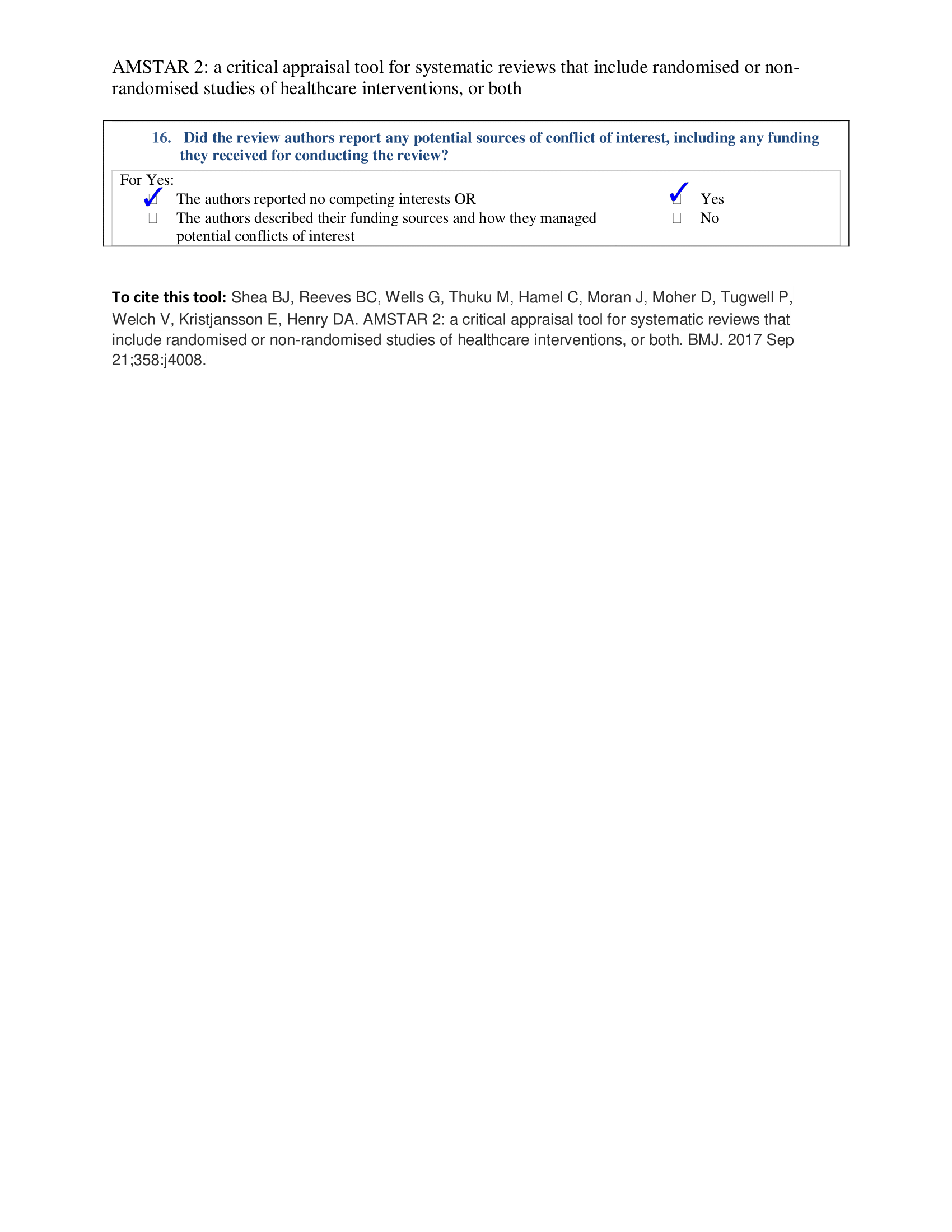


| **Study and Year** | **Fluoroscopy time (minutes), mean (SD) or Median (IQR)** | | **Contrast dye (mL), mean (SD) or Median (IQR)** | | **Total stent length (mm), mean (SD) or Median (IQR)** | | **Vessel diameter (mm), mean (SD) or Median (IQR)** | |
| --- | --- | --- | --- | --- | --- | --- | --- | --- |
|  | **RA** | **FA** | **RA** | **FA** | **RA** | **FA** | **RA** | **FA** |
| **Dall’Ara 2023** | 26 (20-35) *  25 (19-30) ** | 26 (19-33) | 162 (130-210) *  167 (127-190) ** | 160 (129-226) | 48 (30-58) *  31 (18-43) ** | 31 (18-49) | 3.3 (0.5) *  3.3 (0.5) ** | 3.6 (0.6) |
| **Desta 2022** | 30 (21-44) | 28 (21-40) | - | - | - | - | - | - |
| **Ferstl 2022** | 24 (14) | 30 (19) | 217 (91) | 217 (98) | 59 (34) | 62 (34) | - | - |
| **Giustino 2023** | - | - | - | - | - | - | - | - |
| **Januszek 2020** | - | - | 201.9 (80.7) | 223.5 (105.2) | - | - | - | - |
| **Kassimis 2014** | - | - | - | - | 38 (18) | 36 (19) | - | - |
| **Kotowycz 2015** | 43.8 (18.1) | 40.5 (21.2) | 384 (189) | 429 (182) | 34.2 (27.3) | 40.3 (27.9) | - | - |
| **Kubler 2018** | 20 (14-27) | 22 (17-32) | 250 (200-300) | 280 (200-350) | - | - | - | - |
| **Solomonica 2020** | 31 (16) | 29 (14) | 205 (77) | 220 (92) | - | - | - | - |
| **Watt 2009** | - | - | - | - | 38.1 (19.4) | 35.2 (21.3) | - | - |
| **Watt 2017** | - | - | - | - | - | - | - | - |
| **Yin 2015** | - | - | - | - | 24 (19) | 26 (21) | 3.0 (0.5) | 3.1 (0.3) |

FA, femoral access; IQR, interquartile range; mL, milliliter; mm, millimeter; RA, radial access; SD, standard deviation

*radial standard access

**radial sheath less guiding catheter access

**Table S3.** Angiographic data of the patients in the included studies

| **Study** | **Radial Access** | **Femoral Access** |
| --- | --- | --- |
| **Frestl et al. 2022** | 6F (39.5%), 7F (60.5%) | 6F (16.1%), 7F (76.7%), 8F (7.2%) |
| **Solomonica et al. 2020** | 6F (100%) | >6F (79%) |
| **Dall’Ara et al. 2023** | 6F (27.4%), 7F (55.2%), 7.5F (16.6%), 8F (0.9%) | 6F (27.4%), 7F (55.2%), 7.5F (16.6%), 8F (0.9%) |
| **Kassimis et al. 2014** | 7.5F (Sheathless) | 8F |
| **Kotowycz et al. 2015** | 6F (69%), 7F (31%), 8F (0%) | 6F (30%), 7F (62%), 8F (9%) |
| **Kübler et al. 2018** | 6F (default) | 6F (default), 7F (for burrs >1.75mm) |
| **Watt et al. 2009** | 6F (70.7%), 7F (29.3%) | 6F (28.9%), 7F (34.2%), 8F (36.8%) |
| **Watt et al. 2017** | Not Reported | Not Reported |
| **Yin et al. 2015** | 6F (59%), 7F (31%), 8F (10%) | 6F (10%), 7F (78%), 8F (12%) |
| **Desta et al. 2022** | Not Reported | Not Reported |
| **Giustino et al. 2023** | Not Reported | Not Reported |
| **Januszek et al. 2020** | Not Reported | Not Reported |

**Table S4.** Sheath sizes used in radial and femoral access across included studies.

| **Study** | **Anticoagulant Used** | **ACT Target** | **Use of Bivalirudin (Angiomax)** | **Antiplatelet Therapy** | **Glycoprotein IIb/IIIa Inhibitors** | **Meets Criteria (Heparin alone, ACT 250-300s)?** |
| --- | --- | --- | --- | --- | --- | --- |
| **Kassimis et al.** | Unfractionated Heparin (70-100 U/kg) | >300 sec (unless abciximab is used) | Operator discretion | DAPT | Abciximab or Bivalirudin used at the discretion | No (ACT >300s, GPIIb/IIIa allowed) |
| **Desta et al.** | Heparin (91.2%), Bivalirudin (9.2%) | Not specified | Used in 9.2% | Aspirin + Clopidogrel/Ticlopidine (62.9%) or Ticagrelor/Prasugrel (34.2%) | Used in 22.0% | No (Bivalirudin and GPIIb/IIIa inhibitors used) |
| **Kubler et al.** | Heparin (infusion via burr sheath) | >250 sec | Not reported | Aspirin + Clopidogrel (except 3 on Ticagrelor, 1 on Prasugrel) | Not reported | Yes (Heparin alone, ACT >250s) |
| **Solomonica et al.** | Bivalirudin (3.2% Radial, 29% Femoral) | Not reported | Used in 29% (Femoral) | DAPT (96% Radial, 84% Femoral) | Not reported | No (Bivalirudin used) |
| **Giustino et al.** | Not reported | Not reported | Not reported | Not reported | Not reported | No (No anticoagulation details) |
| **Arra et al.** | Not reported | Not reported | Not reported | DAPT pretreatment (30.5%) | Not reported | No (Lacks anticoagulant details) |
| **Yin et al.** | Unfractionated Heparin (70 U/kg IV) | 250 sec (for procedures >1 hr) | Not mentioned | Preloaded with DAPT (Aspirin + Clopidogrel) | Use at the operator’s discretion | Partially (Heparin used, but GPIIb/IIIa inhibitors allowed) |
| **Watt et al. 2017** | Not explicitly mentioned | Not specified | Not mentioned | Likely DAPT (based on high DES use) | Used in 13.7% (Radial) and 14.9% (Femoral) | No (Lacks Heparin details, ACT, and GPIIb/IIIa inhibitor specifics) |
| **Watt et al. 2009** | Unfractionated Heparin (70 U/kg IV) | 250 sec (for procedures >1 hr) | Not mentioned | Preloaded with DAPT | Used in 49.3% (Radial) and 42.1% (Femoral) | Partially (Heparin used, but GPIIb/IIIa inhibitors allowed) |
| **Kotowycz et al. 2015** | Intravenous Heparin | Not specified | Not mentioned | DAPT (Aspirin + Clopidogrel) | Used in 52% (Femoral) and 54% (Radial) | No (Lacks ACT details, Heparin alone not specified) |
| **Frestl et al. 2022** | Not mentioned | Not specified | Not mentioned | DAPT (No specific agents detailed) | Not mentioned | No (Lacks anticoagulant, ACT, and GPIIb/IIIa inhibitor details) |
| **Januszek et al. 2020** | Not mentioned | Not specified | Not mentioned | No details provided (DAPT likely used) | Not mentioned | No (Lacks all anticoagulant details) |

*ACT*, activated clotting time; *DAPT*, dual antiplatelet therapy; *DES*, drug-eluting stent; *GPIIb/IIIa*, glycoprotein IIb/IIIa inhibitors; *IV*, intravenous; *UFH*, unfractionated heparin

**Table S5:** Anticoagulant and antiplatelet strategies used across included studies.

| Study and Year | Selection | | | | Comparability | Outcome | | | NOS score |
| --- | --- | --- | --- | --- | --- | --- | --- | --- | --- |
| Dall’ Ara 2023 | * | * | * | * | * | * | * | * | 8 |
| Desta 2022 | * | * | * | * | ** | * | * | * | 9 |
| Ferstl 2022 | * | * | * | * | * | * | * | * | 8 |
| Giustino 2023 | * | * | * | * | * | * | * | * | 8 |
| Januszek 2020 | * | * | * | * | ** | * | * | * | 9 |
| Kassimis 2014 | * | * | * | - | * | * | * | * | 7 |
| Kotowycz 2015 | * | * | - | * | ** | * | * | * | 8 |
| Kubler 2018 | * | - | * | * | * | * | * | * | 7 |
| Solomonica 2020 | * | * | - | * | * | * | * | * | 7 |
| Watt 2009 | * | * | - | * | * | * | * | * | 7 |
| Watt 2017 | * | * | * | * | ** | * | * | * | 9 |
| Yin 2015 | * | * | * | - | * | * | * | * | 7 |

**Table S6.** Newcastle-Ottawa Quality Assessment for cohort studies.


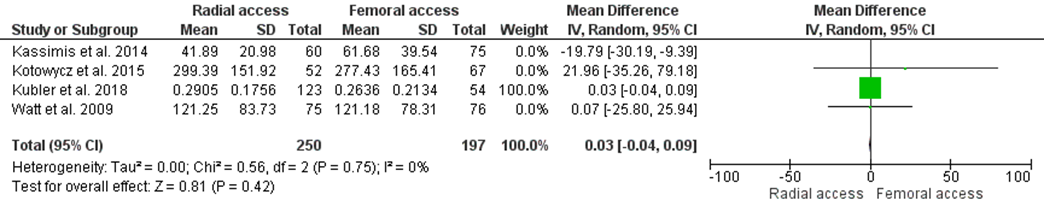


**Figure S3.** Leave-one-out analysis to test the heterogeneity of radiation exposure

**Figure S4.** Funnel plots: **A)** major vascular site bleeding, **B)** procedural success

|  | **Major vascular site bleeding** | **Procedural success** |
| --- | --- | --- |
| Egger p value | 0.00231 | 0.06156 |
| Begg’s p value | 0.27576 | 0.04910 |

**Table S7.** Begg’s and Egger test p values

**Figure S5.** Scatter plots: A) age, B) hypertension, C) diabetes mellitus

**Figure S6.** Scatter plots: A) current smoker, B) BMI, C) hyperlipidemia

**Figure S7.** Scatter plots: A) previous MI, B) previous CABG, C) ejection fraction

**Figure S8.** Scatter plots: A) previous PAD, B) previous Kidney disease, C) male sex %
